# Whole-Genome Sequencing and iPLEX MassARRAY Genotyping Map an EMS-Induced Mutation Affecting Cell Competition in *Drosophila melanogaster*

**DOI:** 10.1534/g3.116.029421

**Published:** 2016-08-29

**Authors:** Chang-Hyun Lee, Gerard Rimesso, David M. Reynolds, Jinlu Cai, Nicholas E. Baker

**Affiliations:** Department of Genetics, Albert Einstein College of Medicine, Bronx, New York 10461

**Keywords:** *Drosophila melanogaster*, whole-genome sequencing, iPLEX MassARRAY, cell competition, Xrp1, Flybook

## Abstract

Cell competition, the conditional loss of viable genotypes only when surrounded by other cells, is a phenomenon observed in certain genetic mosaic conditions. We conducted a chemical mutagenesis and screen to recover new mutations that affect cell competition between wild-type and RpS3 heterozygous cells. Mutations were identified by whole-genome sequencing, making use of software tools that greatly facilitate the distinction between newly induced mutations and other sources of apparent sequence polymorphism, thereby reducing false-positive and false-negative identification rates. In addition, we utilized iPLEX MassARRAY for genotyping recombinant chromosomes. These approaches permitted the mapping of a new mutation affecting cell competition when only a single allele existed, with a phenotype assessed only in genetic mosaics, without the benefit of complementation with existing mutations, deletions, or duplications. These techniques expand the utility of chemical mutagenesis and whole-genome sequencing for mutant identification. We discuss mutations in the *Atm* and *Xrp1* genes identified in this screen.

Forward genetic screens in *Drosophila* remain important for the discovery of gene functions and to the genetic characterization of biological processes (St. Johnston 2002; Wangler *et al.* 2015). Accessible whole-genome sequencing permits rapid identification of newly isolated mutations, and several studies have described whole-genome sequencing approaches to mutation identification in *Drosophila* (Blumenstiel *et al.* 2009; Gerhold *et al.* 2011; Gonzalez *et al.* 2012; Haelterman *et al.* 2014). There are, however, additional steps required between the determination of DNA sequence for a newly mutagenized strain and the final identification of a single mutation responsible for a particular phenotype. One challenge is that *Drosophila* strains are not identical in sequence, even before mutagenesis (Bender *et al.* 1983; Mackay *et al.* 2012). Strain polymorphisms greatly outnumber newly induced mutations when most mutant sequences are compared to the reference. A second challenge is that mutagenesis with the typical 25 mM dose of EMS results in one mutation per 273–480 kb (Cooper *et al.* 2008), corresponding to multiple new mutations on each chromosome.

In principle, conventional genetic mapping should be able to associate a single novel mutation with the novel phenotype (Sarin *et al.* 2008). In practice, *Drosophila* studies often concentrate on loci where multiple alleles have been isolated, and prefer complementation testing with extensive collections of pre-existing mutant lines, deficiencies, and duplications to identify the gene (Venken *et al.* 2010; Bellen *et al.* 2011; Roote and Russell 2012). For example, Blumenstiel *et al.* identified *encore* as a mutated gene responsible for disrupting eggshell chamber morphology using complementation tests with publicly available mutant strains (Blumenstiel *et al.* 2009). Such an approach is not universally applicable, since mutant strains do not yet exist for all *Drosophila* genes. Gerhold *et al.* used genetic mapping to identify loci required for compensatory growth following imaginal disc damage (Gerhold *et al.* 2011). Gonzalez *et al.* recognized *ect4* as a gene required for Wallerian degeneration from its mutation in each of three noncomplementing mutant strains (Gonzalez *et al.* 2012). In an extensive recent study, mutant X-linked loci causing defects in neural development or homeostasis were identified in 274 of 394 sequenced strains using complementation with duplications covering portions of the X-chromosome as a mapping strategy (Haelterman *et al.* 2014), an approach analogous to deficiency mapping for autosomal mutations.

Whole-genome sequencing remains sparingly used for novel mutant identification in *Drosophila*, partly because the approaches described above are not always applicable. For example, it may not always be straightforward to obtain multiple alleles of new loci, particularly for genetic backgrounds that grow poorly or for phenotypes that cannot be assessed rapidly, either of which may limit the size of genetic screens. In addition, recognition of allelism requires complementation analysis, which is also a prerequisite for deletion or duplication mapping, or for complementation with existing mutant strains. Complementation analysis is not straightforward for phenotypes that are only apparent in genetic mosaics. Genetic mosaicism is an important tool in *Drosophila* for investigating function in later developmental stages or adults of genes that are essential early in development. The usual method makes use of inducible FLP recombinase expression to achieve mitotic recombination between homologous chromosomes bearing FRT sites, yielding daughter cells homozygous for particular chromosome arms from heterozygous parents (Xu and Rubin 1993). In this way clones of homozygously mutant cells may be obtained in animals that are largely heterozygous, which enables characterization of the roles of essential genes in particular tissues. Mitotic recombination does not produce cells that are transheterozygotes of different alleles, or of alleles and deficiencies, unless an unlinked rescue transgene or gene duplication is employed, which is usually not feasible for unidentified new mutants.

One process that is only revealed by genetic mosaic studies is cell competition. Cell competition describes the elimination of certain otherwise viable genotypes from genetic mosaics. Cell competition was originally described for the Minute class of mutations (Morata and Ripoll 1975), but has also been described for mosaics of certain other genotypes (Baker 2011; de Beco *et al.* 2012; Vivarelli *et al.* 2012; Levayer and Moreno 2013; Amoyel and Bach 2014). Minutes are dominant slow growing mutations now known to correspond to the majority of the ribosomal protein loci, which affect growth dominantly because of haploinsufficiency (Marygold *et al.* 2007). Minute mutations are typically lethal as homozygotes, but survive as heterozygotes with slow growth and thin or small (“Minute”) bristles (Lambertsson 1998). Despite the viability and fertility of Minute (*i.e.*, *Rp*/+) flies, clones of *Rp*/+ cells are not generally recovered in tissues derived from otherwise wild-type imaginal discs, since they are eliminated by competitive apoptosis (Morata and Ripoll 1975; Simpson 1979; Moreno *et al.* 2002; Kale *et al.* 2015). Because cell competition only occurs in genetically mosaic animals, the effect of mutations on cell competition can only be assessed in genetic mosaics. This makes for a cumbersome phenotypic assay and largely precludes complementation analysis. For example, tumor suppressors of the *Salvador-Warts-Hippo* pathway were recognized in a screen for mutations that are required for cell competition by virtue of their other roles in growth regulation and organismal viability, but mutations that affected cell competition specifically and lacked other apparent phenotypes, although recovered, were not identified (Tyler *et al.* 2007).

Here, we describe a genetic screen for further EMS-induced mutations affecting cell competition, using methods for mutation identification and mapping that avoid genetic complementation assays. We describe a Burrows–Wheeler Aligner/Genome Analysis Toolkit (BWA/GATK) pipeline for the identification of novel mutations from whole-genome sequences that automatically excludes strain differences between the reference genome and the FRT genetic background that is essential for mosaic generation. To permit mapping of a single mutation without recourse to complementation tests, we employ iPLEX MassARRAY genotyping. The iPLEX MassARRAY uses matrix-assisted laser desorption ionization time of flight (MALDI-TOF) mass spectrometry to detect sequence variants. Mass spectrometry is sufficiently quantitative that allele frequencies can be estimated from pools of recombinants, greatly facilitating mapping and reducing its expense (Buetow *et al.* 2001; Ding and Cantor 2003; Ding *et al.* 2004). A flowchart outlining the sequencing and mapping process is shown in Figure 1. These methods expand the utility of whole-genome sequencing to identify single mutations and mutations affecting mosaic traits in *Drosophila*.

**Figure 1:**
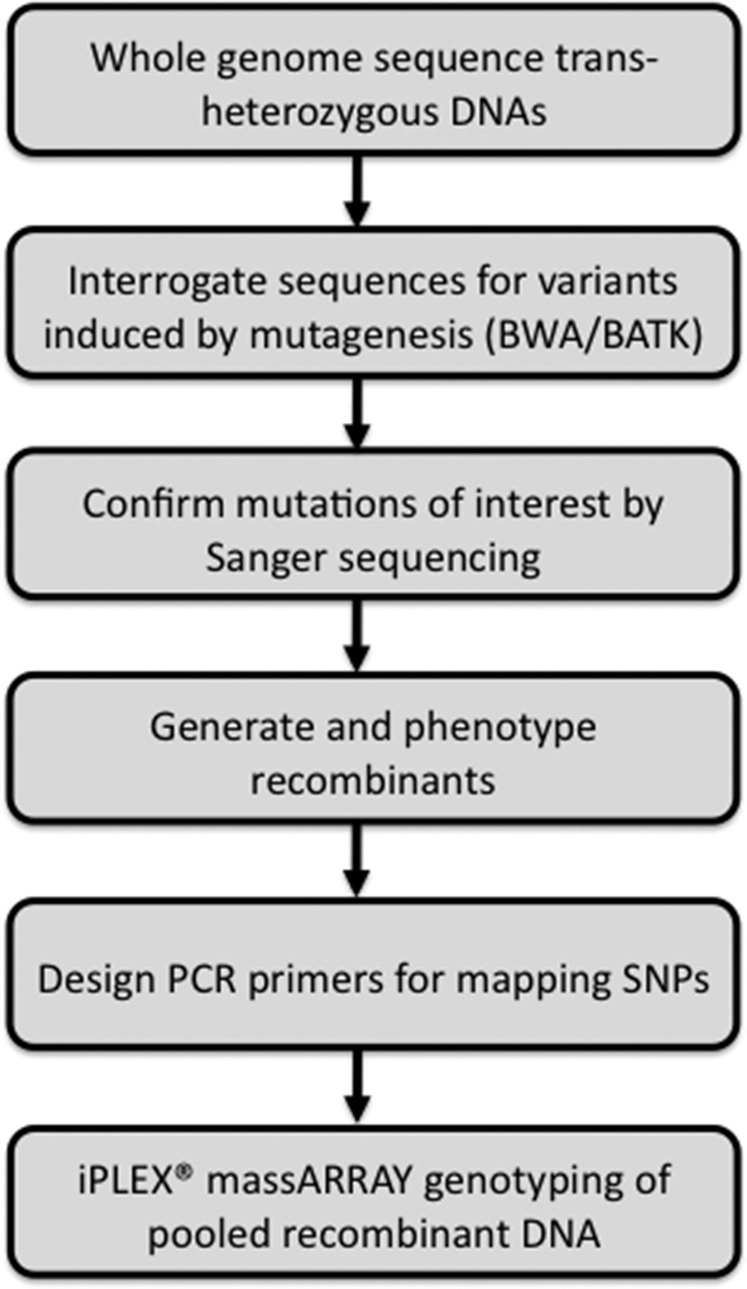
Flowchart of steps leading to identification and mapping of a single mutant allele without recourse to genetic complementation assays or collections of known mutants.

## Materials and Methods

### Fly strains and husbandry

Strains used in this study: FRT82B (Xu and Rubin 1993), P{w, *arm*-LacZ} (Vincent *et al.* 1994), P{w, *tub*-Gal80} (Lee and Luo 1999), *RpS3^Plac92^* (Andersson *et al.* 1994), *Atm*^3^ (Silva *et al.* 2004).

For clones in the adult eye, we used an eye-specific enhancer-induced FLP (*ey*FLP2) insertion on the X chromosome (Newsome *et al.* 2000). For heat-shock-induced clones, we used a FLP122 insertion on the X chromosome (Chou and Perrimon 1996). Larvae were subjected to 60 min at 37°, 72 hr after egg deposition. Dissection and fixation were performed 96 hr after clone induction. All crosses were made at 25° on cornmeal agar medium.

### Mutagenesis and screening

**Figure 2:**
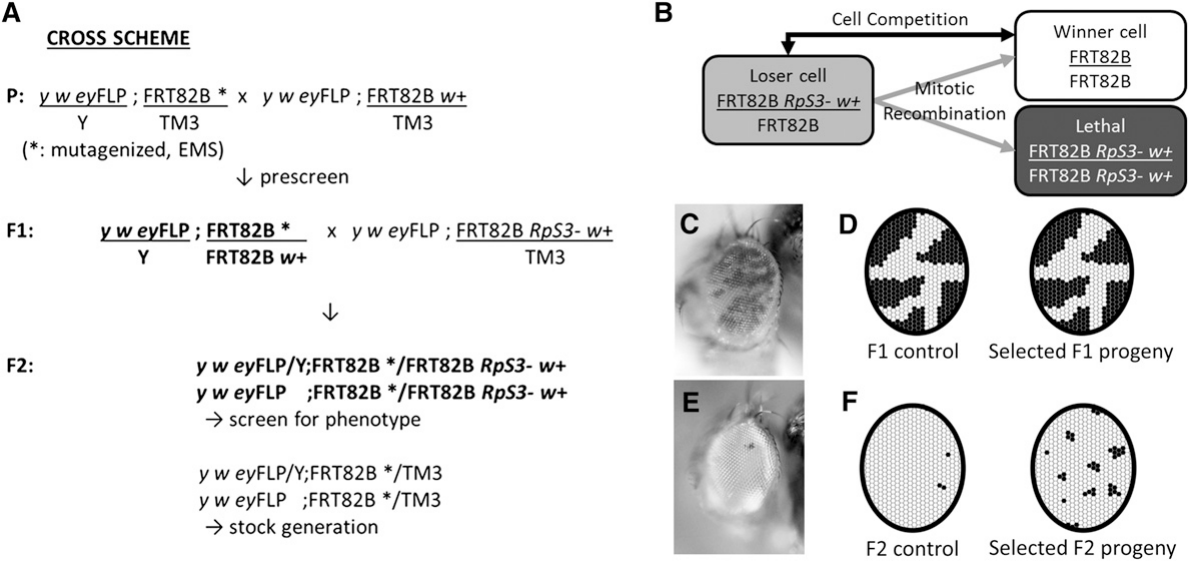
Scheme of a forward genetic screen to find genes required during cell competition. (A) The F2 crossing scheme for screening mutants on chromosomal arm 3R. In the F1 generation, single F1 male progeny with equal contributions of red and white eye tissues were selected (illustrated in C, D). After breeding with *RpS3*^Plac92^ females, the F2 progeny with appropriate genotype were screened for candidate mutant strains (illustrated in E, F). Mutants were recovered and bred from balanced siblings. (B) Cartoon of the screen concept. FLP-FRT mediated mitotic recombination in *RpS3* heterozygous cells produces unpigmented, non-Minute FRT82B homozygous cells and reciprocally recombinant cells homozygous for the mutation in the *RpS3* gene. The latter die, leaving the FRT82B homozygous clones to compete with unrecombined genotype *RpS3* heterozygous genotype. (C) Adult eye image of an unmutagenized control fly in the F1 generation. Genotype: *y w ey*FLP; FRT82B *w*+ *arm*-LacZ/FRT82B (unmutagenized). (D) Schematic of the F1 generation. Mosaic flies that are indistinguishable from controls in proportions of red and white eye cells were selected. These should lack novel mutants that cell-autonomously alter growth, in which the unpigmented clones should either be relatively smaller (in case of a growth deficit) or larger (in case of a mutation causing overgrowth). (E) Adult eye image of an unmutagenized control fly in the F2 generation. Few pigmented cells remain in the eye, which is largely homozygous for the unmutagenized FRT82B progenitor chromosome. (F) Schematic of the F2 screen. Mutants that might affect cell competition are selected from F2 genotypes where more pigmented cells survive than in controls (see Figure 3, B and D for examples). Genotype: *y w ey*FLP; FRT82B/FRT82B P{w+, *arm*-LacZ} P{w+, *tub*-Gal80} *RpS3*.

Male flies from an isogenized stock (*y w ey*FLP; FRT82B/TM3, *Ser*^1^) were fed with 15 mM EMS in sucrose solution as described (Newsome *et al.* 2000). Subsequent crosses were made as shown in Figure 2B. Mutagenized males were crossed to non-Minute *ey*FLP virgins (*y w ey*FLP; FRT82B P{w+, *arm*-LacZ}/TM3, *Ser*^1^) to generate mosaic eyes (Newsome *et al.* 2000). Single F1 males were crossed to Minute virgin females (*y w ey*FLP; FRT82B P{w+, *arm*-LacZ} P{w+, *tub*-Gal80} *RpS3*^Plac92^/TM3, *Ser*^1^) to screen for cell competition inhibiting mutants in the F2 generation as in Figure 1B. Stocks were recovered from the balanced F2 siblings.

### Immunohistochemistry

Imaginal discs immunohistochemistry and confocal imaging was performed as described using paraformaldehyde-lysine-periodate fixation (Baker *et al.* 2014). Primary antibodies used were mouse anti-β-galactosidase (mAb40-1a) obtained from Developmental Studies Hybridoma Bank, and rabbit anti-activated Dcp-1 (Cell Signaling Technology, Danvers, MA). Secondary antibodies were multilabeling antibodies from Jackson Immunoresearch Laboratories (West Grove, PA).

### DNA preparation for sequencing and bulk segregant analysis

Genomic DNA for sequencing was prepared from transheterozygous late third instar larvae grown at 18°, to maximize polytene amplification in which euchromatin is over-represented, as described (Blumenstiel *et al.* 2009).

Genomic DNA for iPLEX MassARRAY was prepared from phenotyped adult mosaics transheterozygous for recombinant and parental FRT82B P{w+, *arm*-LacZ} P{w+, *tub*-Gal80} *RpS3^Plac92^* chromosomes. A total of 50 independent recombinants were pooled from each of the phenotypically wild-type and phenotypically M2-73 populations. Adults were ground in liquid nitrogen using a pestle and mortar, and DNA was recovered using the same procedure as for sequencing.

### Genome sequencing and variant calling

Library construction for whole-genome sequencing was performed according to the manufacturer’s protocol (TruSeq DNA Sample Prep Kit v1, Illumina, San Diego, CA). Whole-genome resequencing was performed on Illumina HiSequation 2000 platform with paired-end 2 × 100 reads (Illumina). Adapter or low quality bases were trimmed from sequence reads. Then the cleared sequences were aligned to the reference genome dm3 using the BWA (Li and Durbin 2009). The Mark Duplicates program from Picard (http://picard.sourceforge.net/) was used to remove duplicate reads. Local realignment, the recalibration of base quality values, and the adjustment of per-base alignment qualities were performed by components from the GATK pipeline (McKenna *et al.* 2010; DePristo *et al.* 2011). The Unified Genotyper of GATK was applied to call the sequence differences or indels. Due to the fairly aggressive manner of the Unified Genotyper in making either SNP or indel calls, the raw call sets was filtered to reduce false-positive results based on call quality, depth, strand bias, *etc*., according to GATK best practice (version 4).

Three mutant strains were exceptional in showing much higher frequencies of sequence differences. In each case there was a single large hypervariable region on chromosome 3R, within which apparent mutations occurred at much higher rate than normal (one mutation per 323 bp). These hypervariable regions were distinct but each partially overlapped another. This suggests that these three mutant chromosomes each contained sequences derived from a common progenitor that differs substantially from both the FRT82B chromosome and the reference genome sequence. We are unsure when these sequences entered our FRT82B strain, but sequences from these three anomalous regions have been excluded from our sequence analysis results.

### Sanger sequencing

Primers of 20–22 bp length and annealing temperature of 57–63° were designed for 500–700 bp PCR products. NCBI/Primer-BLAST was used to optimize primer design and minimize off-target sites. QIAquick PCR Purification Kit (Qiagen) was used to purify PCR products for Sanger sequencing.

### Recombination for mapping

The M2-73 chromosome was allowed to recombine with an isogenized chromosome marked with *Tb*^1^. Recombinant offspring containing *Tb*^1^ and FRT82B were selected by Geneticin (G418) resistance. Single recombinant males were bred with the *y w ey*FLP; FRT82B P{w+, *arm*-LacZ} P{w+, *tub*-Gal80} *RpS3*/TM3, *Ser*^1^ tester strain and the offspring scored as phenotypically either mutant (cell competition defective) or nonmutant (cell competition manifested).

### iPLEX MassARRAY

PCR and extension primers were designed from sequences containing each target SNP and ∼100 upstream and downstream bases with Assay Design Suite (http://agenabio.com/assay-design-suite-20-software) using the default settings. The PCR primers were pooled to a final concentration of 500 nM. The extend oligonucleotides were ranked according to mass and divided into three groups. The lowest mass group was rehydrated to 88 μM, the middle group to 110 μM, and the highest mass group to 165 μM. A 50 μl aliquot from each of the extend oligonucleotides was pooled to make an unextended extend primer (UEP) pool. The PCR primer pool was used to amplify 10 ng of DNA in a 5 μl volume reaction with 1 U of FastStart Taq Polymerase (Roche, Indianapolis, IN) and 4 mM magnesium chloride. The tubes were cycled 45 times with an annealing temperature of 56°. After PCR, shrimp alkaline phosphatase (SAP) was used to dephosphorylate any remaining dNTPs to render them unusable for future polymerase reactions. A total of 0.5 U of SAP and buffer (Agena Bioscience, San Diego, CA) was added to the PCR tubes and incubated for 40 min at 37°, followed by 5 min at 85°. Single base extension reactions were performed on the PCR reactions with the iPLEX Gold Kit (Agena Bioscience) and 0.8 μl of the custom UEP pool. The kit contains mass modified terminator nucleotides that increase the mass difference between extended UEPs, allowing for greater accuracy in genotyping. The mass difference with unmodified terminator nucleotides ranges from 9 to 40 kDa, depending on the two nucleotides compared. With the mass modified terminator nucleotides the mass difference increases to 16–80 kDa. The single base extension reactions were cycled with a nested PCR protocol that used five cycles of annealing and extension nested with a denaturation step in a cycle that was repeated 40 times for a total of 200 annealing and extension steps. The goal was to extend nearly all of the UEPs. Following single base extension, the reactions were diluted with 16 μl of water and deionized with 6 mg of resin. After deionizing for 20 min the reactions were dispensed onto SpectroChip Arrays with a Nanodispenser (Agena Bioscience). The speed of dispensation was optimized to deliver an average of 18 nl of each reaction to a matrix pad on the SpectroChip. An Agena Bioscience Compact Mass Array Spectrometer was used to perform MALDI-TOF mass spectrometry according to the iPLEX Gold Application Guide (Gabriel *et al.* 2009). Availability of MassARRAY Spectrometer facilities can be determined through Agena Bioscience. The Typer 4 software package (Agena Bioscience) was used to analyze the resulting spectra and the composition of the target bases was determined from the mass of each extended oligo.

## Results

### EMS mutagenesis in search for genes required during cell competition

Although a number of genes affecting cell competition have been identified, significant questions remain. An interaction between competing genotypes is thought to trigger cell death only in the loser cells, but the identities of the interacting molecules that initiate cell competition have not been identified. To help address this and other outstanding questions, we conducted a chemical mutagenesis to screen for further genes required during cell competition. The breeding scheme used, as well as the principle of the screen, are described in Figure 2A. We mutagenized a strain with an FRT site near the centromere of chromosome 3R, at position 82B, in order make clones homozygous for this arm. Mutagenized chromosomes were bred with FRT82B P{w+, *arm*-LacZ} P{w+, *tub*-Gal80} *RpS3^Plac92^* strains in a background of the *ey*FLP transgene to obtain flies in which cells transheterozygous for the mutagenized and *RpS3^Plac92^* chromosomes were subject to mitotic recombination in the head (Newsome *et al.* 2000). The *RpS3^Plac92^* mutation is a typical Minute mutation, corresponding to the classically defined gene M(3)95A (Marygold *et al.* 2007). Since the *RpS3^Plac92^* mutation is cell lethal when homozygous (Figure 2B), the resulting adult eyes typically consist almost entirely of recombined FRT82B/FRT82B cells, distinguished by the lack of eye pigment (Figure 2E). We reasoned that the few remaining, pigmented, *RpS3* heterozygous cells have survived two processes: first, they represent the minority of cells where chronic FLP expression did not lead to mitotic recombination before the end of development; second, they survived competition with the increasing population of non-Minute (*RpS3*^+/+^) FRT82B homozygous cells. We hypothesized that mutations that diminished cell competition (or mitotic recombination) would lead to increased representation of unrecombined, pigmented, *RpS3^Plac92/+^* cells in this assay (Figure 2F). Therefore, increased contribution of pigmented cells offers an easily visualized screen for mutations that might affect cell competition. To help discriminate against mutations affecting recombination, or recessively diminishing growth, which could also result in a higher proportion of pigmented cells, an F2 screening procedure was adopted (Figure 2A). In the F1 generation, heterozygotes between mutagenized chromosomes and control chromosomes were screened to identify mosaic flies where the two genotypes were equally represented in the adult eyes, selecting against mutations with effects on recombination or growth (Figure 2, C and D). This procedure should also eliminate any mutations dominantly affecting FLP-mediated mitotic recombination, should such mutations exist. F1 flies satisfying this condition were then individually bred with a FRT82B P{w+, arm-LacZ} *RpS3^Plac92^* strain to identify mutations potentially required for cell competition using the F2 generation.

A total of 2731 such F2 crosses were screened, recovering 19 mutated lines that reproducibly enhanced the contribution of *RpS3* heterozygous cells in the F2 generation without enhancing the contribution of non-Minute cells in the F1 generation. Only one of these mutagenized chromosomes was homozygously viable. Crosses between all the other strains revealed lethal complementation groups of four alleles and two alleles (although these apparent complementation results were misleading, as described in the section on Atm mutations and cell competition and 13 mutant lines that complemented all the other lethal lines.

### Whole-genome sequencing of mutants

We identified the novel mutations in the mutant strains by sequencing the genomes of all 15 distinct complementation groups, as well as the second allele from the two-allele complementation group, for a total of 16 genotypes. Since most mutant homozygotes were lethal, genomic DNA from heterozygotes was sequenced. To facilitate the differentiation of new mutations induced by chemical mutagenesis from strain polymorphisms between the reference genome and the FRT82B chromosome that was necessary in this mosaic screen, we adapted a transheterozygote sequencing approach pioneered by Gerhold *et al.* (Gerhold *et al.* 2011). For example, to identify the mutations present in four mutant strains M2-73, M9-22, M9-25, and M10-26, whole-genome sequencing was performed on the four transheterozygous genotypes M2-73 +/+ M9-22, M9-22 +/+ M10-26, M10-26 +/+ M9-25, and M9-25 +/+ M2-73. Interpretation of these transheterozygous sequences is described below.

Sequencing was performed on an Illumina HiSeq2000 sequencing platform, as two groups of eight sequences, each with multiplexed 100 bp paired-end reads in a single lane platform (see *Materials and Methods* for details). One group of eight samples was then resequenced in a further lane platform, which doubled the read depth for these samples.

### Identification of mutations after whole-genome sequencing

We used BWA, which is fast and accurate, to align sequences (Li and Durbin 2009). BWA was employed to align sequence output to the *Drosophila melanogaster* genome version dm3 (+BDGP Release 5) (Hoskins *et al.* 2007). Since Illumina HiSeq2000 can generate up to 35 Gbp of sequence per lane, and the *Drosophila* genome sequence comprises 180 Mb (Adams *et al.* 2000), we anticipated around 24× read depth for eight samples sequenced from a single lane, and 48× read depth for eight samples sequenced in two lanes. About 23× and 50× read depth were actually achieved (Table 1). A further statistic that is useful when assessing the reliability of heterozygous mutant detection is the percentage of the genome sequenced to ≥10× read depth. Surprisingly, this was ∼95% in both cases (Table 1). This relative independence from overall read depth suggests that the remaining 5% of the genome for which <10× coverage is available may represent problematic sequences, for example repetitive regions that are not aligned well, and that additional sequencing might yield higher overall read depth but little more information in such difficult regions.

**Table 1:** Sequencing statistics

	Mean Target Coverage	% of Target Bases ≥10×	Total Variants on 3R		Protein Coding Variants on 3R	
			No Filter	RD ≥ 15, GQ ≥ 30	No Filter	RD ≥ 15, GQ ≥ 30
One Lane Sequences	22.7 (±6.2)	94.90 (±4.37)	310.2 (±68.2)	83.7 (±25.3)	23.8 (±6.1)	12.5 (±4.8)
Two Lane Sequences	49.7 (±8.2)	96.50 (±0.08)	229.4 (±39.7)	139.6 (±25.4)	21.8 (±0.4)	16.4 (±0.9)
*P* Value (*t* Test)		ns	*P* < 0.05	*P* < 0.001	ns	*P* < 0.05

Mutation predictions for 16 mutants strains sequenced in either one or two Illumina lanes, as indicated. Results are reported prior to selective validation by Sanger sequencing, and include false positives. RD (read depth) and GQ (genome quality) represent filters for sequence quality. See Results section on identification of mutations for details. Errors represent ± 1 SD.

Gerhold *et al.* were the first to exploit transheterozygote sequencing in this way, using a customized Perl script to identify induced mutations (Gerhold *et al.* 2011). We were able to use the publicly available Unified Genotyper of GATK for this purpose (McKenna *et al.* 2010; DePristo *et al.* 2011). The algorithm compares four transheterozygous sequences to identify heterozygous variants that are detected in exactly two sequences out of four. This procedure automates the removal of strain differences between the FRT82B background and the reference genome, which are expected both to be homozygous and present in all four samples, and the removal of the large majority of alignment errors, since these will usually be distributed randomly among the four sequences (regions of inconsistent alignment, for example those related to indels or repeats, may lead to mixed base calls that resemble heterozygous variants in a single sequence). The procedure should also select against variants from regions that appear “difficult to sequence,” and produce high false-positive mutation rates (Haelterman *et al.* 2014). Specifically, our algorithm for each mutant calls for 0/1 in the two relevant sequences, and 0/0 in the remaining two samples, where “0” corresponds to reference allele and “1” to any variant. In case a novel mutation affected a site already differing between the reference sequence and the FRT82B strain, we also allowed for a 1/2 combination in two sequences with a 1/1 combination in the remainder, where “2” represents another distinct allele. At least regarding mutations in coding regions, our primary interest, no such event was observed.

Initially, filters were set to prioritize regions of more reliable sequence. We required a threshold of read depth ≥15, genotype quality ≥30 (a Phred-scaled confidence measure for the reported genotype) (McKenna *et al.* 2010; DePristo *et al.* 2011). These analyses found, on average, 83.7 (±25.3) candidate mutations on chromosomes arm 3R for the once-sequenced samples, and 139.6 (±25.4) for the twice-sequenced samples, of which 12.5 (±4.8) and 16.4 (±0.9) were the average numbers of nonsynonymous mutations affecting coding regions (Table 1; errors are ±1S.D.).

To evaluate the accuracy of the variant calls, genomic DNA was amplified from transheterozygote genomic DNA samples and subject to Sanger sequencing. From 16 candidate coding mutations identified from the samples sequenced in a single Illumina lane, and 21 candidate coding mutations predicted from the samples sequenced twice, each one was confirmed by Sanger sequencing.

Given the high apparent accuracy observed when sequence quality filters were applied, whereby no false positives were observed, sequences were re-evaluated without read depth or genotype quality filters, identifying an average 226.5 additional, lower confidence variants from samples sequenced once, of which 11.3 were nonsynonymous coding mutations, and 89.8 additional variants in samples sequenced twice, of which 5.4 were nonsynonymous coding mutations (Table 1). A sample of these lower-confidence mutations were checked by amplification and Sanger sequencing. Out of 16 lower confidence coding mutations predicted from the samples sequenced in a single Illumina lane, Sanger sequencing confirmed 14. Out of another 18 lower confidence coding mutations predicted from the samples sequenced twice, 16 were confirmed.

Taken together, these data indicate that even without applying filters for sequence quality, the false-positive rate of mutant identification is low. If the results are pooled as if no sequence filters had been applied from the outset, the measured false-positive rates for predicted coding mutations were 5.9% for samples sequenced to 23× read depth and 2.8% for samples sequenced to 50× read depth. Filtering for genome quality eliminated false positives, but appeared to conceal about one-third of the mutations present in samples sequenced to 50× read depth, and the majority of those present in the once-sequenced samples, because many fewer mutations were identified when filters were applied (Table 1). Without filters, the overall number of coding mutations identified is very similar in the samples of different read depth. An average of 23.8 coding mutations were predicted from samples sequences in one lane, of which 5.9% are expected to be false positives, and 21.8 predicted from samples sequenced in two lanes, of which 2.8% are expected to be false positives. Similar mutation recovery independent of read depth may argue that false-negative detection rates are also low (see *Discussion*). However, there was an effect of read depth on recovery of noncoding mutations (Table 1).

G/C-to-A/T transitions accounted for 74.4% of the predicted coding mutations, in agreement with previous studies of EMS mutagenesis in *Drosophila* (Winkler *et al.* 2005; Cooper *et al.* 2008; Blumenstiel *et al.* 2009). The results indicate that one mutation occurred every 149 kb in 3R euchromatin, more than predicted by earlier whole-genome sequencing studies (Table 2) (Blumenstiel *et al.* 2009; Gerhold *et al.* 2011), which is a further indication of improved mutation detection.

**Table 2:** Frequency of mutations

	Blumenstiel *et al.* (2009)*^a^*	Gerhold *et al.* (2011)	1 Lane (23× Coverage)	2 Lanes (50× Coverage)
EMS Concentration	45	25	15	15
Mutation Rate (kb/Mutation)	273	97	94.0 (±22.4)	124.6 (±21.8)
False-Positive Rate (%)	17.6	<25	5.3*^b^*	2.8*^b^*

A comparison of the mutation rates (nonparental single nucleotide variants) reported in different studies as kb/mutation. Errors represent ± 1 SD. We found a higher mutation frequency than Blumenstiel *et al.* (2009), and a similar frequency to that reported by Gerhold *et al.* (2011; on a different chromosome arm) reported using mutagenesis with 25 mM EMS, a higher concentration than that used in our study. As recorded in Table 1, when total variants were recorded the frequency of variants predicted decreased significantly (*P* < 0.05) after deeper sequencing, suggesting that there may be a higher frequency of false positives when sequencing depth is lower, although this was not seen for coding variants. Mutation frequencies for our study and the Gerhold *et al.* (2011) study were calculated using the +BDGP release five reference sequence (dm3) that was the alignment template for all these studies.

a Based on 70.9% of the genome sequenced after filtering.

b These false-positive rates were determined for coding variants (see Results section on identification of mutations).

### Atm mutations and cell competition

The M9-25 mutant strain, one of the four-member complementation group, contained a mutation in the Ataxia telangiectasia mutated (*Atm*) gene, a locus important in DNA repair (Abraham 2001). Unexpectedly, the other three noncomplementing strains all harbored the identical allele, as did two other mutants that had been sequenced at the whole-genome level, M2-17 and M10-22. This mutation, a C to A alteration at position 15,241,548 (+BDGP Release 6) generated a premature stop codon predicted to truncate the 2767 amino acid Atm polypeptide after residue 162. Despite the presence of the identical *Atm* mutation, M9-25, M2-17, and M10-22 each showed a typical spectrum of mutations, distinct from one another. A total of 39 of the 52 coding mutations observed in these strains reflected alkylation events typical for EMS mutagenesis. By contrast, the 15,241,548 C to A transversion is not typical of EMS. These data argue against the accidental isolation of the same EMS-induced allele multiple times, and suggest that a spontaneous *Atm* mutant allele was segregating in the isogenized FRT82B progenitor strain and was recovered on multiple occasions after passing through the mutagenesis.

**Figure 3:**
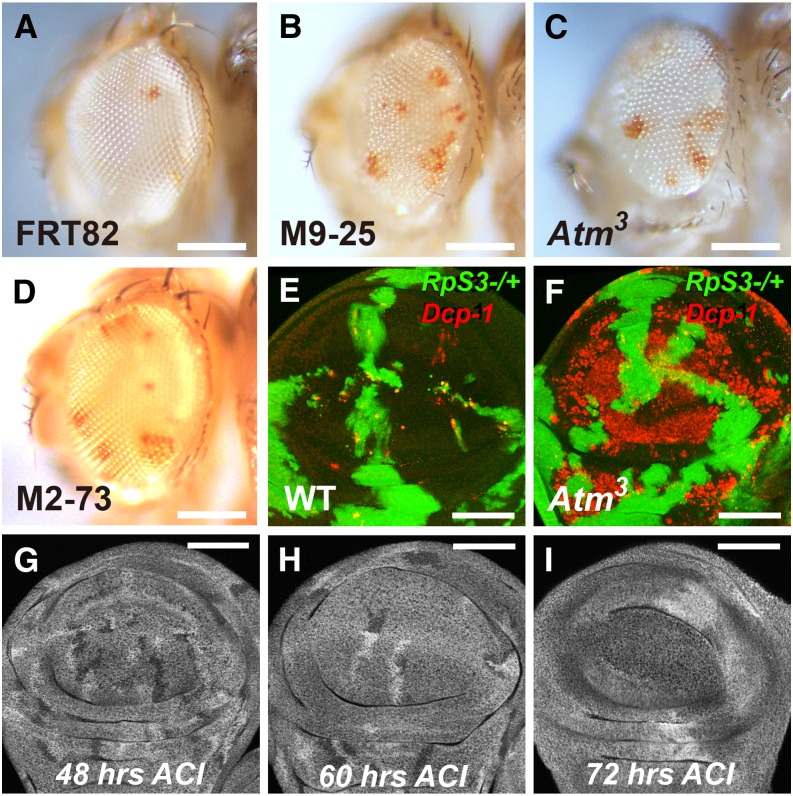
*Atm* and *Xrp1* mosaic phenotypes in Minute and non-Minute backgrounds. (A–D) Adult eye phenotypes from F2 screen (see Figure 2A for method and genotypes) (A) FRT82B control; (B) FRT82B M9-25; (C) FRT82B *Atm*^3^; (D) FRT82B M2-73. (B–D) Homozygous M9-25, *Atm*^3^, and M2-73 cells were less able to eliminate pigmented *RpS3* heterozygous cells. (E, F) Late third instar wing imaginal disc. The *RpS3*/+ background is labeled for β-galactosidase expression in green; non-Minute clones are unlabeled. Cell death is detected with α-activated-*Dcp*-1 labeling in red. (E) Competitive cell death of *RpS3*/+ cells is largely limited to boundaries between the genotypes. (F) High cell death levels were observed throughout *Atm*^3^ clones. (G–I) *Atm*^3^ homozygous clones induced between 48 and 72 hr in a non-Minute background and dissected at late third instar larval stage from 48 to 72 hr after clone induction (ACI) as indicated. The FRT82B *Atm*^3^ heterozygous background was labeled for β-galactosidase expression, the FRT82B homozygous twin-spots were labeled more brightly, the FRT82B *Atm*^3^ homozygous clones were unlabeled. *Atm*^3^ homozygous clones were under-represented compared to twin-spots after 72 hr but not after shorter times. Scalebars represent 250 microns for panels A–D and 50 microns for panels E–I. Genotypes: (A) *y w ey*FLP;FRT82B/FRT82B P{w+, *arm*-LacZ} P{w+, *tub*-Gal80} *RpS3*, (B) *y w ey*FLP;FRT82B M9-25/FRT82B P{w+, *arm*-LacZ} P{w+, *tub*-Gal80} *RpS3*, (C) *y w ey*FLP;FRT82B *Atm*^3^/FRT82B P{w+, *arm*-LacZ} P{w+, *tub*-Gal80} *RpS3*, (D) *y w ey*FLP;FRT82B M2-73/FRT82B P{w+, *arm*-LacZ} P{w+, *tub*-Gal80} *RpS3*, (E) *hs*FLP;FRT82B/FRT82B P{w+, *arm*-LacZ} P{w+, *tub*-Gal80} *RpS3*, (F) *hs*FLP;FRT82B *Atm*^3^/FRT82B P{w+, *arm*-LacZ} P{w+, *tub*-Gal80} *RpS3*, (G–I) *y w hs*FLP;FRT82B *Atm*^3^/FRT82B P{w+, *arm*-LacZ}.

To verify that the mutation in *Atm* was responsible for the phenotype, the independent *Atm*^3^ mutation was studied. The *Atm*^3^ mutation corresponds to a premature stop codon in place of amino acid 600 (Pedersen *et al.* 2010). Mosaic Minute eyes containing homozygous *Atm*^3^ clones showed an increased contribution of *RpS3* heterozygous cells in the adult; in addition, the mosaic adult eyes were slightly small and rough in all of these mutants (Figure 3, A–C).

To understand the effect of *Atm* mutations, homozygous *Atm*^3^ clones were induced in *RpS3* heterozygous wing discs using *hs*FLP. An antibody against the activated caspase *Dcp*-1 revealed a strikingly high level of apoptosis throughout the *Atm*^3^ clones (Figure 3, E and F), consistent with a previous report of spontaneous apoptosis in *Atm*^−/−^ cells in the wing imaginal disc (Oikemus *et al.* 2004). This may explain the retention of *RpS3* heterozygous cells in our screen. Chronic apoptosis can reduce clonal growth (Shi *et al.* 2003), so reduced growth of homozygous *Atm*^3^ clones likely leads to a greater proportion of the eye being descended from the remaining *RpS3* heterozygous cells. A plausible explanation for why a mutation deleterious for clonal growth was not excluded in the F1 generation was suggested by inducing *Atm*^3^ homozygous clones in a wild-type background at different time points (Figure 3, G–I). When induced 48 or 60 hr before dissection in the later third instar, *Atm*^3^ homozygous clones were present at sizes and numbers similar to the reciprocal recombinant twin clones in wing imaginal discs. *Atm*^3^ homozygous clones were only under-represented when dissected 72 hr after clone induction (Figure 3I). This suggests that *Atm*^3^ homozygous clones survive without significant growth effects for many hours, only later reducing in viability (Figure 3H). The *Atm*^3^ homozygous clones seen in eyes of the F1 generation of our screen may have been induced by *ey*FLP too late to exhibit strong growth effects in a non-Minute background, by contrast to the *RpS3^Plac92^/+* F2 generation that grew for ∼2 d longer.

Two alleles of a second putative lethal complementation group were sequenced. Both mutants, however, showed an identical spectrum of novel mutations on chromosome 3R, indicating that they represented reisolation of the same mutant, presumably induced premeiotically. Therefore, the specific mutation responsible for the phenotype could not be revealed as a locus independently mutated in the two lines.

### Mapping a single allele using recombination and iPLEX MassARRAY

None of the other 13 mutant strains shared any alleles that affected the open reading frames of common loci. In addition, no mutations were observed in genes yet implicated in cell competition. Since each mutant strain contained mutations affecting ∼20 protein coding regions on chromosome 3R, as well as many alterations in noncoding regions, other methods were needed to identify the responsible genes. We decided to focus on the M2-73 mutant, which gives a clear and reproducible phenotype in the mosaic eye assay (Figure 3D).

In other genome sequencing projects, identification of causative mutations has been facilitated by complementation with existing alleles (Zhai *et al.* 2003). Complementation is difficult to apply to phenotypes scored in mosaics. It would be possible to test existing mutations for similar phenotypes to M2-73, but loss-of-function mutations existed for only five out of the 22 coding mutations in M2-73 and many are not recombined onto FRT82B chromosomes and carry transgenic *w*+ alleles that would interfere with the adult eye assay. Finally, there is no guarantee that existing alleles would present identical phenotypes to uncharacterized missense mutations present on the M2-73 chromosome. Software prediction of which amino acid substitutions affect protein function using SIFT (http://sift.jcvi.org/) (Kumar *et al.* 2009) predicted that 17 of the 22 nonsynonymous mutations present on M2-73 would be deleterious, doing little to prioritize likely mutations.

Recombinational mapping required phenotypic scoring and genotyping of recombinant chromosomes in mosaic flies for the phenotypic assessment of recombinant chromosomes. We applied iPLEX MassARRAY to map the mutation responsible for the M2-73 phenotype, an approach that is both generally applicable, accurate, efficient, and economical. In iPLEX MassARRAY, a primer that precisely flanks a mutant site is extended from an amplified genomic DNA template using a mass modified dideoxynucleotide, then MALDI-TOF mass spectrometry was used to determine the allele (Buetow *et al.* 2001; Ding and Cantor 2003). The accuracy of mass spectrometry permits measurement of an allele frequency from pooled samples, and multiple SNPs can be assessed simultaneously if their specific primers differ in mass (Ding *et al.* 2004).

We first analyzed amplified DNA from FRT82B M2-73/FRT82B P{w+, *arm*-LacZ} P{w+, *tub*-Gal80} *RpS3^Plac92^* transheterozygotes to confirm heterozygosity for 11 novel SNPs identified after the mutagenesis (the identity of all 236 novel SNPs recovered on the M2-73 chromosome is shown in Supplemental Material, Table S1). These were distributed across 3R at 1.6–2.6 Mb intervals. Ideally, the peaks for wild-type and mutant products from heterozygous DNA should be equal. We found nine loci where wild-type and mutant signals were within 10% of one another, one locus where only the wild-type allele was detected, and another that yielded 80% wild-type product (Figure S1). Such deviations from equality can occur if either chromosome differs from the reference sequence used to guide primer design, so that the primers do not amplify each genotype equally.

**Figure 4:**
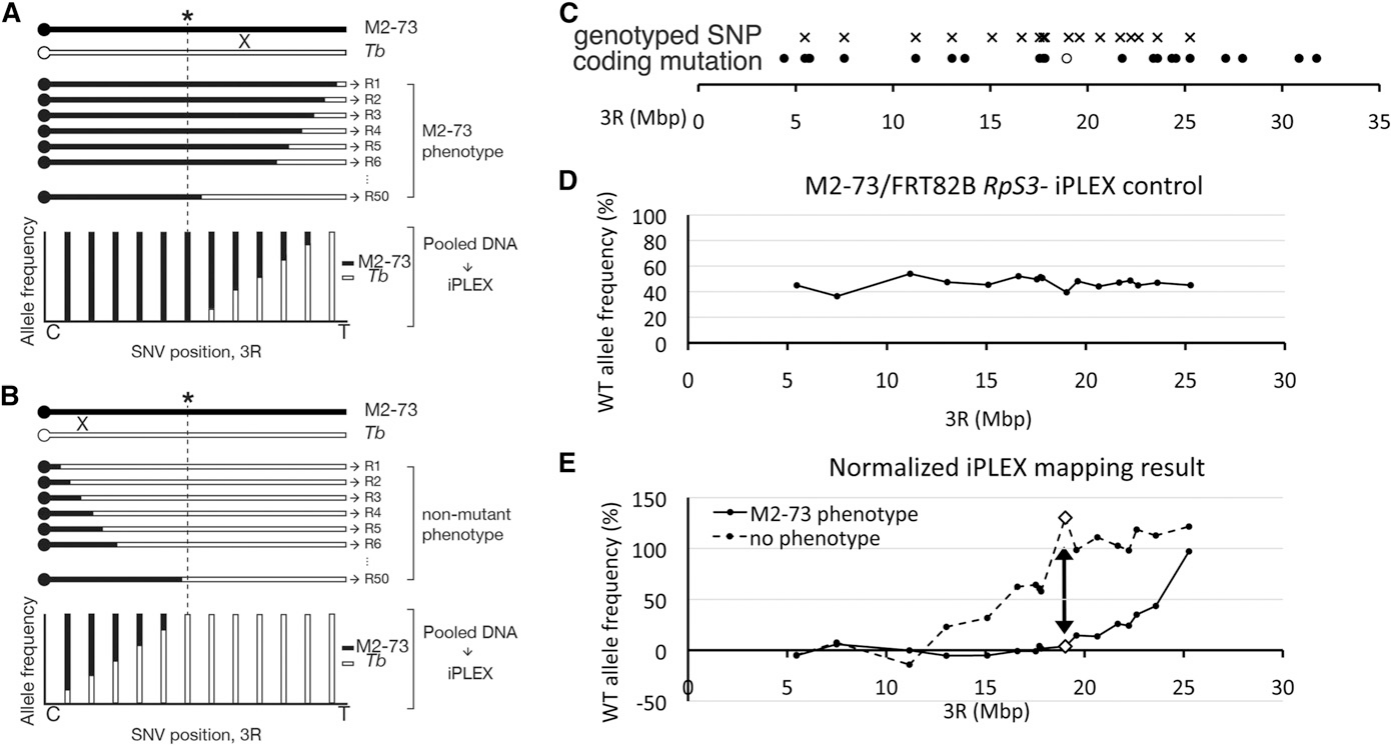
iPLEX MassARRAY genotyping maps the M2-73 mutant. (A, B) Recombinant mapping principles. ‘X’ illustrates that meiotic recombination occurs between homologs. (A) A cartoon representation of recombinant chromosome arms 3R expected to retain the M2-73 phenotype. These should retain M2-73 SNPs proximal to and including the locus responsible (asterisk), and exhibit various crossover points distal to the locus. When DNA from 50 such recombinants is pooled, the frequency of M2-73 SNPs is expected to decrease distal to the locus as the frequency of *Tb* SNPs increases. (B) A cartoon representation of recombinant chromosome arms 3R expected to lack the M2-73 phenotype. These should retain *Tb* SNPs distal to and including locus responsible for the M2-73 phenotype, and exhibit crossover points proximal to the locus. When DNA from such recombinants is pooled, the frequency of M2-73 SNPs should increase proximally to the locus as the frequency of *Tb* SNPs decreases. Comparing observed SNP frequencies for the two pools should limit the mutant locus to a chromosome segment that retains 100% M2-73 SNPs in phenotypically mutant recombinants and 100% *Tb* SNPs in phenotypically wild-type recombinants. (C) Distribution of the 17 SNPs that were assessed by iPLEX (crosses) and 22 exonic variants on the M2-73 chromosome (circles). An open circle shows the position of the coding mutation in Xrp1, closed circles show the other mutations. Some of the coding alleles were used as SNPs, in addition to noncoding SNPs. (D) Allele frequencies determined by iPLEX for 17 SNPs in the FRT82B M2-73/FRT82B P{w+, *arm*-LacZ} P{w+, *tub*-Gal80} *RpS3* genotype. Deviation from 50% reflects amplification and detection bias in the iPLEX method (see section on Mapping a single allele using recombination and iPLEX MassARRAY). This heterozygote genotype is used because the recombinant DNA is also heterozygous with the FRT82B P{w+, *arm*-LacZ} P{w+, *tub*-Gal80} *RpS3* chromosome, in which background the M2-73 phenotype is assessed. (E) Normalized frequencies of reference alleles (derived from the *Tb*^1^ chromosome). Solid line, pooled recombinants exhibiting the M2-73 phenotype; dashed line, pooled recombinants lacking the M2-73 phenotype. Normalized allele frequency is calculated as (measured % *Tb* allele in recombinants)/(measured % *Tb* allele in (D) control) × 100, to account for any amplification or detection bias in the MassARRAY method. The arrow indicates SNP 19,029,906 (+BDGP Release 6) where *Tb* SNPs are recovered in ∼0% of phenotypically M2-73 recombinants and ∼100% of phenotypically wild-type recombinants. This SNP is ∼100 kb from a coding mutation in the *Xrp1* gene (open circle in A).

Genetic recombinants were obtained after breeding FRT82B M2-73 with an isogenized *Tb*^1^ strain. The dominant marker *Tb*^1^ maps distal on chromosome 3R, distant from FRT82B. Recombinant FRT82B *Tb*^1^ strains were assessed in mosaics for presence of the M2-73 phenotype, and DNA extracted from a pool of 50 independent recombinants exhibiting the M2-73 phenotype and 50 independent recombinants exhibiting the control phenotype. Allele frequencies were determined for each pool by iPLEX MassARRAY. It is expected that the mutant allele should be present in all the recombinants exhibiting the mutant phenotype, and none of the recombinants lacking the phenotype (Figure 4, A and B, respectively). In this initial experiment, the pool of phenotypically nonmutant recombinants exhibited only *Tb*^1^ alleles from the distal tip of 3R to 19,588,401 bp (+BDGP Release 6, cytological band 92A10), and the pool of phenotypically mutant recombinants exhibited only *M2-73* alleles from the proximal end of 3R to 17,720,801 bp (+BDGP Release 6, cytological band 90C2). This indicates the causative mutation mapped to the interval 17,720,801–19,588,401 bp (Figure S1).

In a refinement step, eight additional polymorphic sites within the critical region were assessed with the same recombinant pools. The combined results with all 17 polymorphisms are shown in Figure 4E. The combined results show that the pool of phenotypically wild-type recombinants exhibited only wild-type alleles from the distal tip of 3R to the SNP at 19,029,906 bp (91E4), whereas the pool of phenotypically mutant recombinants exhibited only mutant alleles from the proximal end of 3R to the SNP at 19,029,906 bp. This indicates that the mutation causing the M2-73 phenotype mapped close to 19,029,906 bp (+BDGP Release 6, 91E4); the results exclude loci left of 17,807,692 bp, where some phenotypically wild-type recombinants have M2-73 alleles, and right of 19,588,401 bp, where some phenotypically mutant recombinants have wild-type alleles (Figure 4E). Among the 22 nonsynonymous coding mutations on the right arm of the M2-73 third chromosome, one satisfied these criteria. This was the G to T transition in the second exon of the *Xrp1* gene (position 18,925,491 bp, +BDGP Release 6), which predicted a nonsense mutation in the open reading frame (Figure 4C).

## Discussion

We applied whole-genome sequencing and iPLEX MassARRAY mapping strategies to mutations with potential effects on cell competition, a process occurring in genetic mosaics. Molecular identification of a mutation with one allele was possible without use of complementation tests. These methods may be useful for mutations responsible for other phenotypes in genetic mosaics or that are otherwise problematic in more conventional approaches.

Genome sequencing has advanced significantly since first applied to *Drosophila* mutants. The Illumina HiSeq2000 platform achieves 35 Gb of sequence per lane. Our experience suggests that 35 Gb of sequence is sufficient to identify coding mutations from at least eight mutant strains. We did not see much increase in the rate of coding mutation identification from a further doubling of read depth. In practice, the proportion of the genome adequately covered (∼95%) increased little with greater read depth, suggesting that the easily sequenced (or easily assembled) genome regions have already been adequately covered. Haelterman *et al.* reported similar conclusions (Haelterman *et al.* 2014).

We adapted a simple yet effective strategy, first implemented by Gerhold *et al.* (Gerhold *et al.* 2011), whereby sequencing transheterozygotes between mutant strains automates the removal of strain polymorphisms from the analysis. It is now possible to implement this strategy using publicly available software. Using this method, none of 65 mutations we confirmed by Sanger sequencing represented a polymorphism between FRT82B and reference strains. Under our least stringent sequence analysis, false-positive rates due to other causes were only 2.8–5.9%, depending on read depth. By contrast, a recent study that sequenced balanced mutant stocks in a conventional manner required three additional filter steps after variant calling to eliminate various other sources of variation. These were: removing polymorphisms distinguishing the FRT and reference genes; removing polymorphisms segregating in the progenitor strain; filtering genes that repeatedly yield false-positive results, at the end of which only 1% of the initial sequence variants remained as candidate novel mutations (Haelterman *et al.* 2014).

Our results suggest little reason to impose sequence quality filters to identify coding mutations. Although unfiltered sequence led to more false positives, it permitted the recovery of significantly more mutations, especially at lower read depth (an average of 9.9 more verified coding mutations confirmed at lower read depth, 4.8 more coding mutations confirmed at higher read depth).

We do not have a direct estimate of the false-negative rate, *i.e.*, mutations that were not detected by whole-genome sequencing. When the estimated false-positive rate is subtracted, we estimate that on average 22.4 novel 3R coding mutations would be confirmed among samples at 23× coverage, and 21.2 among samples at 50× coverage. The failure of increased coverage to identify more mutations is consistent with a low false-negative rate. We also notice that the mutation frequency in our study was one mutation per 94.0–124.6 kb (corresponding to 8.0–10.6 mutations per Mb), which is as high or higher than in previous studies, even though we employed a lower mutagen concentration (Table 2). This suggests that earlier studies might have had higher false-negative rates, perhaps as a result of lower read depths and application of filters to eliminate the large number of background sequence variants. If these interpretations are valid, then following the procedures outlined in this paper should identify EMS-induced mutations with reasonably high accuracy and completeness.

The data in Table 1 suggest that prediction of noncoding variants may be less accurate than for coding variants. Because increased read depth resulted in a significant reduction in the total number of sequence variants predicted on 3R when no sequence quality filters were applied, ∼90% of which are noncoding, it seems likely that 50× coverage significantly reduces false positives in noncoding sequences. We performed too few Sanger sequencing validations of noncoding mutations to quantify this (five noncoding mutations were sequenced and verified in the course of designing primer pairs for iPLEX mapping). Reasons that higher false-positive rates might be expected in noncoding sequences include reduced sequence complexity and more repeated sequences in noncoding DNA.

We have isolated 13 mutants that reduce the elimination of *Rp*/+ cells from mosaic eye discs and identified two of these loci, *Atm* and *Xrp1*. These loci were identified following whole-genome sequencing through distinct approaches. Multiple *Atm* mutants identified a lethal complementation group, so it was evident that the locus in common between the strains was likely responsible, as was confirmed by replicating the phenotype with a pre-existing allele. Such approaches often permit the identification of mutants after whole-genome sequencing (Blumenstiel *et al.* 2009; Gerhold *et al.* 2011; Haelterman *et al.* 2014). Other mutations did not fall into lethal complementation groups, however, and genome sequencing identified no other mutant loci in common. Since the cell competition phenotype can only be assessed in genetic mosaics, approaches based on collections of deficiencies, duplications, or pre-exiting mutations were not appropriate. The remaining strategy was to use recombination to map phenotypes to intervals containing only one mutation. A limitation then is how many recombinant chromosomes must be both genotyped and assessed phenotypically in genetic mosaics. In principle, the results could also have been obtained using DNA sequencing of individual PCR products to characterize recombinants. To save time and expense, we exploited iPLEX MassARRAY methods to map cell competition phenotypes in an efficient and economical manner. iPLEX MassARRAY provides a way to genotype multiple SNPs in pools of recombinants in a single step, without additional sequencing reactions. This approach may be generally useful in mutation mapping following genome sequencing, especially mutations were allelism is not established and where phenotypic evaluation may be time-consuming. iPLEX MassARRAY technology is widely used in human genetics and available in many medical schools, as well as from the spectrometer manufacturer (Agena Bioscience). Mapping could also have been conducted using Sanger sequencing for genotyping, but this might have required thousands of sequencing reactions.

Mutations in *Atm* appear to select for *Rp*/+ cells in the mosaic eyes by eliminating *Atm*^−/−^ cells through a mechanism independent of cell competition. Elevated cell death in *Atm* mutant cells occurs due to chromosome segregation defects including telomere fusion and anaphase bridges (Oikemus *et al.* 2004). Although we aimed to avoid mutations with competition-independent effects on growth through a prescreen of mosaic flies at the F1 generation (Figure 2, A, C, and D), perdurance may have allowed the *Atm* mutation to pass this filter: *Atm* homozygous mutant clones are only lost after 72 hr of growth in wing imaginal discs. Because the F1 generation screened *Atm* homozygous clones in a non-Minute background, in which larval growth is completed ∼2 d earlier than in the Minute background where the effect of cell competition is assessed, the F1 generation may not provide a sufficiently stringent assay to identify mutations that affect noncompetitive growth but exhibit significant perdurance effects.

A mutation in *Xrp1*, by contrast, remains a candidate to affect cell competition. *Xrp1* encodes a DNA-binding bZIP protein whose expression is induced in response to irradiation and other stresses (Brodsky *et al.* 2004; Akdemir *et al.* 2007; Gruenewald *et al.* 2009; Vermeulen *et al.* 2013). *Xrp1* is not required for cell or organismal viability (Akdemir *et al.* 2007). We expect to describe the role of *Xrp1* in cell competition in more detail elsewhere.

Identification of newly-induced point mutations following genetic screens can be a bottleneck, especially when multiple alleles are not obtained or when complementation analyses with pre-existing mutant, deficiency or duplication collections are inconvenient. We present evidence that whole-genome sequencing and variant mapping methods are adequate to identify even single alleles of nonessential genes.

## Supplementary Material

Supplemental Material
