# Supplementary material for: Whole-Genome Sequencing and iPLEX MassARRAY Genotyping Map an EMS-Induced Mutation Affecting Cell Competition in Drosophila melanogaster
Source: G3 (Bethesda). 2016 Aug 29;6(10):3207–17. doi: 10.1534/g3.116.029421 (PMC5068942; doi:10.1534/g3.116.029421)
Supplement: Supplemental Material [file supp_6_10_3207__index.html]

Whole-Genome Sequencing and iPLEX MassARRAY Genotyping Map an EMS-Induced Mutation Affecting Cell Competition in Drosophila melanogaster — Supplemental Material 

# Whole-Genome Sequencing and iPLEX MassARRAY Genotyping Map an EMS-Induced Mutation Affecting Cell Competition in *Drosophila melanogaster*

## Supplemental Material for Lee, *et al*, 2016

**Files in this Data Supplement:**

- Table S1 - Mutations on the M2-73 chromosome. (.pdf, 225 KB)
- Figure S1 - Rough mapping of M2-73. (.tif, 337 KB)
