# Supplementary material for: Whole-Genome Sequencing and iPLEX MassARRAY Genotyping Map an EMS-Induced Mutation Affecting Cell Competition in Drosophila melanogaster
Source: G3 (Bethesda). 2016 Aug 29;6(10):3207–17. doi: 10.1534/g3.116.029421 (PMC5068942; doi:10.1534/g3.116.029421)
Supplement: Supplemental Material [file supp_g3.116.029421_TableS1.pdf]

Nearest Gene	Position of first affected base (dm3, +BDGP Release 5)		Reference allele	M2-73 allele
CG9853	225260	exonic	C	T
Karybeta3	478577	exonic	C	T
CG9769(dist=1161),Nep2(dist=4441)	541079	intergenic	A	T
laf(dist=18914),Gnf1(dist=9178)	723169	intergenic	AC	-
Cdep	739543	intronic	T	C
CG12162	1293555	UTR5	C	T
CG12162	1293804	exonic	C	T
CG2182	1482663	exonic	G	A
cas	1540780	exonic	C	T
CG1213	1670267	UTR5	C	T
CG34113	1721515	intronic	C	T
CG34113	1759141	intronic	C	T
Gasp	1916463	intronic	GGGGG	-
CR43487(dist=16330),CG31559(dist=5831)	1970007	intergenic	C	G
CR43487(dist=16374),CG31559(dist=5787)	1970051	intergenic	T	G
pb	2540921	intronic	C	T
pb	2546021	intronic	C	T
pb	2552104	intronic	C	T
Antp	2725936	intronic	T	A
gfzf	2971904	UTR3	-	T
rn	3107401	intronic	TT	-
rn	3120765	intronic	G	A
CG42656	3318847	exonic	C	T
alpha-Est9	3326499	intronic	T	A
CG34127	3464407	intronic	T	-
CG7918	3893648	intronic	C	T
stck	4181938	upstream	A	T
CG42796	4413751	intronic	G	T
CG8861	5131508	intronic	A	G
CG12951	5155076	exonic	C	T
Teh1	5664303	intronic	G	A
Cyp12e1(dist=52162),hth(dist=20981)	6314588	intergenic	C	T
CG34304	6485686	intronic	A	T
CG4073(dist=7083),tomboy20(dist=12364)	6548704	intergenic	T	C
CG14692	6624796	exonic	T	A
Ugt86Dg	6991157	exonic	C	T
scpr-A	7067546	exonic	A	C
CR43283(dist=5290),CG31386(dist=7990)	7087135	intergenic	T	-
CR31386	7114355	ncRNA_intronic	G	A

pros	7194791	intronic	C	T
pros	7204868	intronic	G	T
CG17230,sals	7277135	intronic	T	G
dpr4	7366533	intronic	C	T
Csk	7473937	intronic	C	T
CG6959	7685751	intronic	T	A
CG6959(dist=2470),sad(dist=5304)	7697653	intergenic	C	T
Tk	7824003	intronic	T	A
Tk(dist=1119),KLHL18(dist=1519)	7832313	intergenic	G	C
dpr15	7983267	intronic	C	T
CG12360	8855443	exonic	T	A
CG8795(dist=4490),CG8784(dist=1310)	9164375	intergenic	T	G
CG8795(dist=4492),CG8784(dist=1308)	9164377	intergenic	A	T
yellow-e(dist=3714),Ir87a(dist=1275)	9244720	intergenic	-	TTT
CR17025(dist=50882),CG12538(dist=5963)	9342710	intergenic	C	T
sqd	9461430	UTR3	C	T
flfl	9516871	exonic	C	T
f-cup(NM_001104302:exon11:c.1570+1G>A)	9522158	splicing	C	T
CG34383	9555776	intronic	AA	-
foxo	9891161	intronic	C	T
foxo	9894633	intronic	G	T
NK7.1	10175674	UTR5	TTCGTTT	-
eff	10565610	intronic	C	T
btsz	10672825	intronic	C	T
btsz(dist=2268),CG17304(dist=3133)	10681894	intergenic	C	T
CG3837(dist=24364),CG14861(dist=68102)	10772390	intergenic	C	T
CG6966	10917764	intronic	C	T
CG18516(dist=54064),CG5302(dist=26047)	11432558	intergenic	T	C
alpha-Man-IIb	11636076	intronic	T	C
alpha-Man-IIb	11638939	intronic	C	T
Akt1(dist=8129),Sb(dist=16083)	11938368	intergenic	C	T
tara	12085124	exonic	C	T
gish	12106865	intronic	T	C
Gyc-89Da	12300066	exonic	C	T
CG42342	12330162	intronic	T	A
CG42342	12387772	intronic	C	T
CG12783	12438631	intronic	C	T
Fas1	12459192	intronic	-	AA
abd-A	12639159	intronic	C	T
msa	12714205	ncRNA_intronic	C	T
msa	12739737	ncRNA_intronic	C	T
Abd-B	12769839	UTR5	G	A
CG8907	12835080	intronic	-	A

Mur89F(dist=31852),CG31262(dist=25316)	13017522	intergenic	A	-
beat-IIb(dist=34080),CG31418(dist=1839)	13107678	intergenic	G	A
CR43490(dist=27900),beat-IIa(dist=3281)	13142440	intergenic	C	T
Sur-8	13226189	intronic	C	T
Dscam3	13311395	intronic	C	T
CG5840	13361974	exonic	T	A
CG7606(dist=1389),sll(dist=7796)	13424028	intergenic	C	T
Pxt	13546523	exonic	C	T
CG7357	13633414	exonic	C	T
Rim	13700747	UTR3	GT	-
Rim	13715688	intronic	C	A
CG42457,cpo	13786879	intronic	C	T
cpo	13842839	UTR3	-	AA
CG7794(dist=17887),htl(dist=1949)	13868987	intergenic	A	-
sr(dist=11147),CG14316(dist=7223)	13970618	intergenic	AA	-
fru	14328221	intronic	C	T
fray	14414725	UTR3	-	AA
CG14304	14442241	intronic	-	TTT
CG34282(dist=6757),VAChT(dist=2919)	14528255	intergenic	G	C
Cha	14550803	intronic	C	T
CG7720	14593268	intronic	A	C
CG31226	14676688	downstream	C	T
CG14298(dist=1870),CG14297(dist=19045)	14704798	intergenic	C	T
Xrp1	14751213	exonic	G	T
CG42613	14761313	intronic	G	-
CG42613	14784129	UTR5	AA	-
gukh	14823055	intronic	C	T
P5cr	14855628	exonic	C	T
cdi	14880956	intronic	AAAAACA	-
CG31475	15024903	intronic	C	T
CG14280(dist=2091),CR43451(dist=26384)	15087695	intergenic	-	AA
Dl	15136668	intronic	C	T
Dl	15151328	UTR5	TGTGTG	-
CG42793(dist=4996),Ino80(dist=9115)	15185602	intergenic	-	T
Dys	15414123	intronic	C	T
Naam	15524594	downstream	C	T
CG31459(dist=1825),CG4662(dist=3453)	15669592	intergenic	C	T
Hs6st	15770004	intronic	TT	-
Hs6st	15820884	intronic	C	T
Hs6st	15833422	intronic	C	T
CR42836(dist=16208),CG5023(dist=13307)	15981851	intergenic	C	T
CG42801,mun	16263200	intronic	TGTTG	TAGTTG
mun	16282181	intronic	T	C
CG4288(dist=12960),MtnE(dist=8872)	16351129	intergenic	C	T

GluRIIE	16470314	UTR3	C	T
CG42322	16579024	intronic	T	C
TotZ	16702894	upstream	C	T
CR43453(dist=14621),CG7922(dist=1212)	17306835	intergenic	T	G
C15	17335220	intronic	C	T
CG31176	17498870	intronic	T	A
CG31176	17525573	intronic	C	T
CG31176	17533636	intronic	A	T
CG31176	17533638	intronic	C	G
Rpl12	17603143	exonic	C	T
CG6678(dist=2988),CG6690(dist=1646)	17735892	intergenic	T	G
CG17843(dist=3926),Eip93F(dist=29642)	17745194	intergenic	C	T
Eip93F	17801426	UTR5	A	T
SKIP	18058695	intronic	-	AAAC
SKIP	18076512	intronic	TT	-
SKIP	18097849	intronic	C	T
CG7084	18125649	intronic	G	C
CG5380	18192234	exonic	C	T
loco	18451232	intronic	C	T
CG42686	18487544	intronic	T	C
CG7029	18617740	intronic	C	T
CG13829(dist=4083),CG13836(dist=2981)	18991143	intergenic	T	A
cnc	19013806	exonic	C	T
CG13826,cnc	19051363	intronic	C	T
pnt	19152413	intronic	C	T
CG42828(dist=1425),CG4393(dist=1545)	19201388	intergenic	-	A
CG4393	19205576	exonic	C	T
CG16732	19360633	UTR3	G	A
CG16732	19360636	UTR3	G	T
CG16732	19360644	UTR3	-	CCT
CG16732	19360653	UTR3	G	T
CG16723	19419430	exonic	C	T
sba	19728350	intronic	C	T
Gdh	19767892	intronic	T	G
CG6364	20116728	exonic	C	T
BRWD3	20146407	exonic	C	T
CG5728	20155938	exonic	T	A
CG6454	20177334	exonic	C	T
CG5789	20366501	exonic	C	T
Esyt2	20373080	intronic	G	C
CG13624	20404724	intronic	C	T
tok	20561347	intronic	C	T
Ast	20588817	intronic	C	T
CG13639,CG31105	20686200	upstream	C	T

CG13646(dist=10247),tnc(dist=24598)	20785508	intergenic	-	GGG
CG11859	21091753	exonic	C	T
Dhap-at	21311750	UTR3	C	T
Nf1	21817722	intronic	T	A
CG33970	22185424	intronic	C	T
CG6490	22357857	exonic	T	A
CG6490	22359257	intronic	C	T
CG14247(dist=5410),CG42789(dist=39606)	22569725	intergenic	C	T
Tl	22629349	intronic	ACAC	-
Lerp	22683019	intronic	C	T
sda	22756554	intronic	A	T
CG31075	22813825	intronic	A	T
CG31075;CG31076	22814423	upstream;downstream	G	-
TwdlC	22819808	upstream	-	C
eater	22922838	exonic	T	A
Tusp	23444104	intronic	C	T
CG12880	23514153	intronic	-	AA
CG12880	23518778	downstream	G	A
CG34353	23614819	exonic	T	C
CG34353	23719663	intronic	A	-
CG4849	23791450	exonic	C	T
CG34362	23884030	intronic	C	T
CG34362(dist=23308),CG34354(dist=37754)	23934792	intergenic	C	T
CG34354	24020685	intronic	-	CACACA
CG34354(dist=14165),CG43125(dist=9340)	24079117	intergenic	A	G
CG34354(dist=14166),CG43125(dist=9339)	24079118	intergenic	T	G
larp	24158516	intronic	C	T
Or98b(dist=17480),beat-VI(dist=47222)	24196704	intergenic	C	T
CG12413(dist=2670),fkh(dist=33611)	24373194	intergenic	A	G
CG14061(dist=25895),CG34295(dist=5487)	24609851	intergenic	GG	-
Doa	24722056	intronic	C	T
CG14521	24758553	intronic	-	CC
CG14521	24766319	intronic	C	T
Ptp99A	25205232	intronic	C	T
Ptp99A	25250954	intronic	-	T
Ptp99A	25284708	intronic	C	T
Trc8(dist=6349),CG2014(dist=5667)	25335868	intergenic	A	C
alph	25432194	intronic	TTTG	-
alph	25440444	intronic	C	T
DopR2	25461861	intronic	C	T
DopR2	25483192	intronic	-	ACAC
Ice	25626875	UTR3	T	A
neo	25650196	exonic	C	T
CG31036	25734736	intronic	C	T

CG31036	25734739	intronic	T	A
Takr99D	25795298	upstream	C	A
CG9747	26015220	intronic	C	T
CecC(dist=5302),CG43448(dist=14855)	26047961	intergenic	T	-
Fer2LCH(dist=18778),CG2217(dist=9405)	26235084	intergenic	C	T
tmod	26399361	intronic	C	T
CG34433(dist=21818),Spn100A(dist=17968)	26498398	intergenic	C	T
CR43238(dist=11297),zfh1(dist=7457)	26584191	intergenic	C	T
Cpr100A	26694389	exonic	C	T
5-HT7(dist=3079),CG33773(dist=15420)	26845648	intergenic	T	A
Gycbeta100B	26962505	intronic	C	T
Gycbeta100B	26977003	intronic	TT	-
CG18672	27086714	exonic	C	T
CG11333(dist=4799),mey(dist=30316)	27294645	intergenic	C	T
mey(dist=24553),nyo(dist=11485)	27357269	intergenic	C	A
mey(dist=24555),nyo(dist=11483)	27357271	intergenic	T	G
nyo	27378387	intronic	C	T
CG2135	27596217	exonic	C	T
sip3	27600505	UTR3	-	AA
heph	27698606	intronic	C	T
heph	27783497	intronic	-	TTT
heph	27802266	intronic	C	T
