# Supplementary figures and images for: Whole-Genome Sequencing and iPLEX MassARRAY Genotyping Map an EMS-Induced Mutation Affecting Cell Competition in Drosophila melanogaster
Source: G3 (Bethesda). 2016 Aug 29;6(10):3207–17. doi: 10.1534/g3.116.029421 (PMC5068942; doi:10.1534/g3.116.029421)

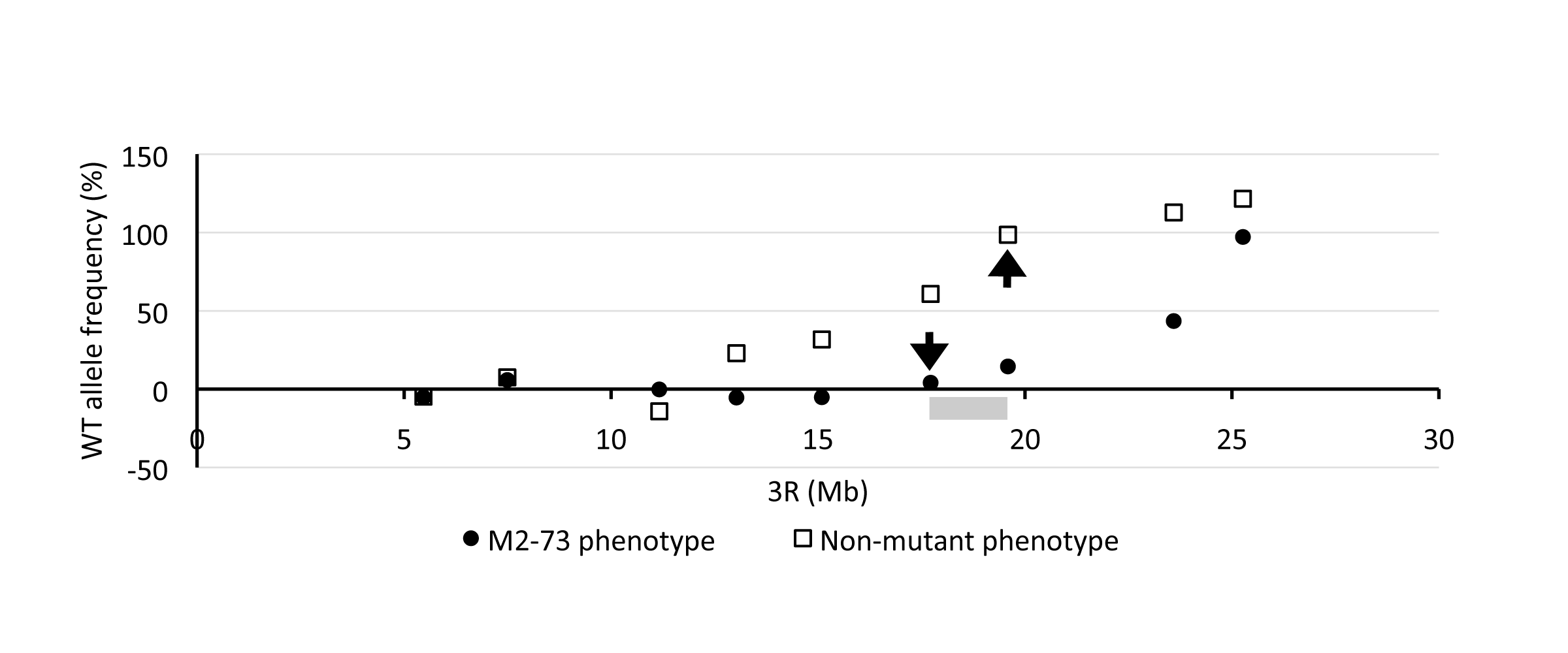

Supplement: Supplemental Material [file supp_g3.116.029421_FigureS1.tif]
